# Dopamine neuron induction and the neuroprotective effects of thyroid hormone derivatives

**DOI:** 10.1038/s41598-019-49876-6

**Published:** 2019-09-20

**Authors:** Eun-Hye Lee, Sang-Mi Kim, Chun-Hyung Kim, Suvarna H. Pagire, Haushabhau S. Pagire, Hee Yong Chung, Jin Hee Ahn, Chang-Hwan Park

**Affiliations:** 1 Hanyang Biomedical Research Institute, Hanyang University, Seoul, 04763 Korea; 2 Paean Biotechnology, Inc., Daejeon, 34028, Korea; 3 Department of Chemistry, Gwangju Institute of Science and Technology, Gwangju, 61005 Korea; 4 Department of Microbiology, College of Medicine, Hanyang University, Seoul, 04763, Korea; 5 Graduate School of Biomedical Science and Engineering, Hanyang University, Seoul, 04763 Korea

**Keywords:** Stem-cell differentiation, Stem-cell research

## Abstract

Parkinson’s disease (PD) is a neurodegenerative disease characterized by progressive movement disturbances caused by the selective loss of dopamine (DA) neurons in the substantia nigra. Despite the identification of the causal mechanisms underlying the pathogenesis of PD, effective treatments remain elusive. In this study, we observed that a low level of fetal bovine serum (FBS) effectively induced DA neurons in rat neural precursor cells (NPCs) by enhancing nuclear receptor-related 1 protein (NURR1) expression. Among the various components of FBS, the thyroid hormones triiodothyronine (T3) and thyroxine (T4) were identified as key factors for the induction of DA neurons. Since an overdose of thyroid hormones can cause hyperthyroidism, we synthesized several thyroid hormone derivatives that can partially activate thyroid hormone receptors and induce the complete differentiation of NPCs into DA neurons. Two derivatives (#3 and #9) showed positive effects on the induction and maturation of DA neurons without showing significant affinity for the thyroid hormone receptor. They also effectively protected and restored DA neurons from neurotoxic insults. Taken together, these observations demonstrate that thyroid hormone derivatives can strongly induce DA neuron differentiation while avoiding excessive thyroid stimulation and might therefore be useful candidates for PD treatment.

## Introduction

Parkinson’s disease (PD) is a progressive neurodegenerative disorder caused by the selective loss of dopamine (DA) neurons in the substantia nigra (SN)^1,2^. Several medications and treatments for improving the symptoms of PD, such as L-3,4-dihydroxyphenylalanine (L-DOPA), dopamine agonists, and deep brain stimulation (DBS), are available. However, the pharmacological treatments currently available can induce side effects such as dyskinesia, and DBS can lead to infections, stroke, or brain hemorrhage^3–11^.

Fetal bovine serum (FBS) collected from the blood of bovine fetuses contains essential components for cell growth, such as growth factors, attachment factors, iron transporters, vitamins, amino acids, cytokines, hormones, proteins, and lipids^12,13^. In neural precursor cell (NPC) cultures, FBS drives NPCs toward an astrocytic fate and interferes with differentiation to a neuronal fate^14^. Among the components of FBS, the hormones triiodothyronine (T3) and thyroxine (T4) promote oligodendrocyte differentiation and myelination^15^.

The relationship between thyroid hormones and DA neurons is well established. For instance, hypothyroidism often leads to the loss of DA neurons^16^. Decreased thyroid hormone levels in the serum induce neurodegenerative changes in the midbrains of *Girk2* mutant mice. The low plasma levels of thyroid hormone in these mutant mice are associated with the downregulation of transforming growth factor alpha (TGF-α) expression in the striatum^17^. Recent studies have demonstrated that thyroid hormones induce the differentiation of mouse DA neurons from embryonic ventral midbrain (VM) NPCs. This suggests that thyroid hormones bind to thyroid hormone receptor α1 (TRα1) to upregulate orthodenticle homeobox 2 (OTX2), which subsequently upregulates NURR1, the transcription factor for dopaminergic differentiation^18^. However, the TRα1-responsive element is not detected within the *Otx2* gene promoter region. Therefore, the relationships among TRα1, OTX2, and NURR1 remain unclear.

In this study, we observed that a low concentration of FBS induced DA neuron differentiation and maturation via an increase in *Nurr1* expression. We demonstrated that the thyroid hormones present in FBS induced DA neurons in rat and human NPCs. We found that only a small amount of the thyroid hormones in FBS were needed to drive DA neuron differentiation in cell cultures. To enable the use of thyroid hormones as a treatment for PD without inducing hyperthyroidism, we developed novel derivatives of thyroid hormones that have a low affinity for the thyroid hormone receptor but can increase the differentiation of DA neurons. These derivatives also had protective effects on DA neurons after neurotoxic insult. We determined the relationship between thyroid hormones and DA neurons, and our observations may assist in the development of new medications for PD.

## Results

### FBS induces differentiation of DA neurons

**Figure 1:**
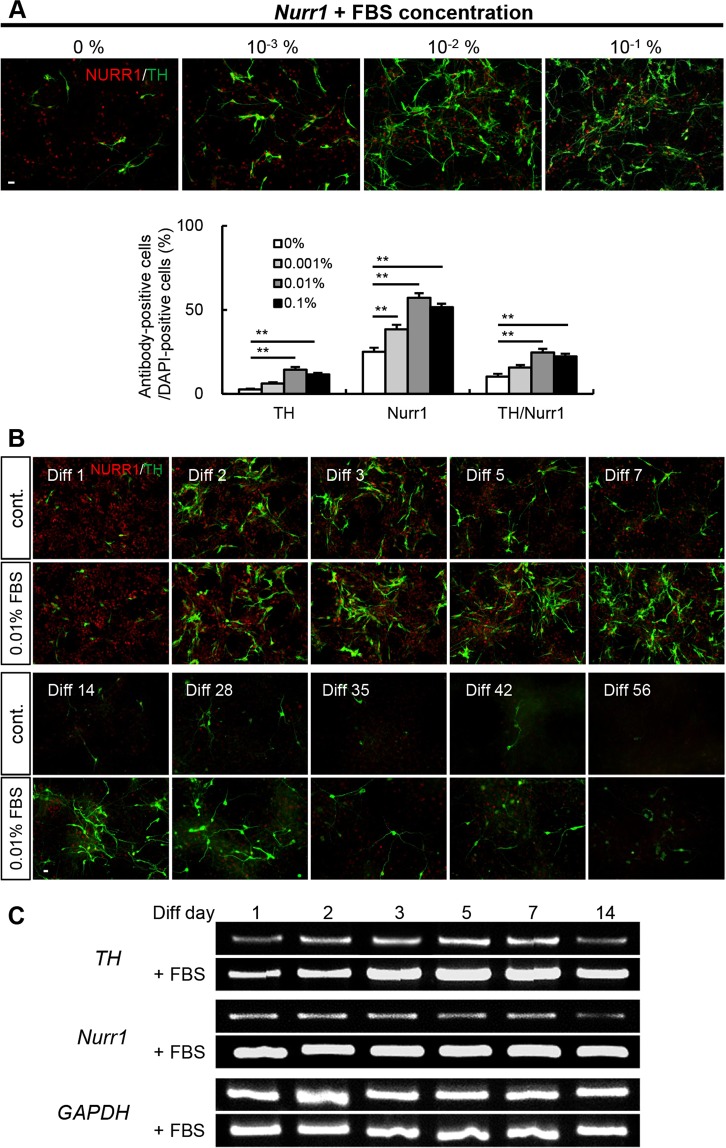
FBS induces DA neuron differentiation and maturation. (**A**) Rat embryonic E14 cortical NPCs treated with FBS for 7 days were differentiated into DA neurons using a *Nurr1*-expressing retrovirus. The differentiated DA neurons were immunostained with anti-NURR1 and anti-TH antibodies, and the number of TH+/DAPI+, NURR1+/DAPI+, and TH+/NURR1+ cells were quantified. (**B**) Rat cortical NPCs treated with FBS were differentiated into DA neurons using a *Nurr1-*expressing retrovirus. As differentiation progressed, FBS-treated cultures showed a dramatic increase in the number of NURR1+ and TH+ cells. The comparisons were performed 8 weeks into the differentiation process. (**C**) RT-PCR analysis for *TH* and *Nurr1* mRNA by differentiation day (days 1–14). The expression of DA neuron–related genes differed between control- and FBS-treated cultures as differentiation progressed. *GAPDH* expression was used as a loading control. **p < 0.01. Scale bar, 20 μm. The error bars are the S.E. from three independent experiments.

NURR1, an orphan nuclear receptor, is expressed predominantly in mesencephalic dopaminergic neurons and is essential for the development of dopaminergic neurons^19^. Because the exogenous expression of NURR1 can robustly increase the proportion of tyrosine hydroxylase (TH)+ neurons after *in vitro* differentiation, rat E14 cortical NPCs were transduced with a *Nurr1*-expressing retrovirus before differentiation. We investigated the effects of FBS on DA neuron differentiation. Treatment with FBS increased the number of TH+ and NURR1+ neurons in a concentration-dependent manner (Fig. 1A; control: 2.7 ± 1.2% [TH/DAPI], 25.1 ± 5.2% [NURR1/DAPI]; 0.001% FBS: 6.2 ± 1.6% [TH/DAPI], 38.4 ± 6.0% [NURR1/DAPI]; 0.01% FBS: 14.4 ± 3.6% [TH/DAPI], 57.1 ± 6.4% [NURR1/DAPI]; 0.1% FBS: 11.6 ± 2.1% [TH/DAPI], 51.6 ± 4.2% [NURR1/DAPI]). Moreover, the percentage of cells coexpressing TH and NURR1 followed a similar pattern (Fig. 1A; control: 10.3 ± 3.8%; 0.001% FBS: 15.7 ± 2.9%; 0.01% FBS: 24.6 ± 5.2%; 0.1% FBS: 22.3 ± 3.4%). We used 0.01% FBS for the rest of our experiments because concentrations >0.01% did not provide any additional advantage for DA neuron differentiation. In addition, we observed that DA neurons persisted for longer durations following the treatment of NPCs with 0.01% FBS. DA neurons persisted for different times beginning 5 days after differentiation, as shown by the histogram in Fig. 1B. On the 56th day after differentiation, only the FBS-treated cells contained DA neurons, whereas no DA neurons remained in the nontreated cells (Fig. 1B). In agreement with the immunostaining results, the mRNA levels of *Nurr1* and *TH* also increased over time in the presence of FBS (Fig. 1C). Taken together, these findings suggest that FBS provides beneficial effects for both the differentiation and maintenance of DA neurons by increasing NURR1 expression.

To test the effects of sera from various species on DA neuron differentiation, we used normal goat serum (NGS), adult rat serum (ARS) and serum replacement (SR). Treating rat cortical NPCs with sera from different species, similar to treating them with FBS treatment, resulted in a greater number of NURR1+ and TH+ neurons than that in the control group. Therefore, treatment with sera may have beneficial effects on DA neuron differentiation regardless of species (Supplementary Fig. 1).

### FBS promotes neuronal differentiation in the presence of NURR1

**Figure 2:**
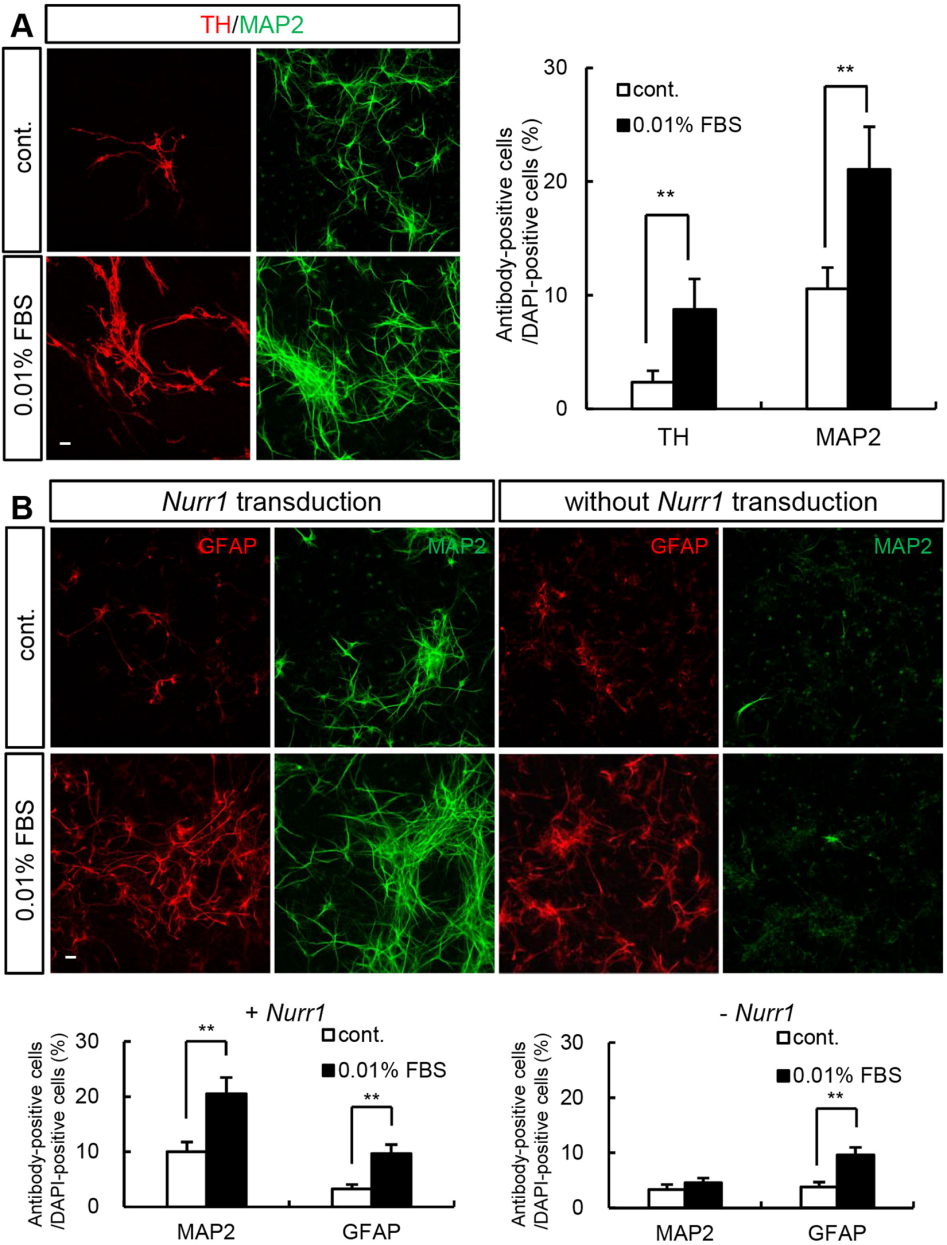
The neurogenic effect of FBS is NURR1-dependent. (**A**) To test whether FBS induces general neurogenesis, the expression of the neuronal-specific marker MAP2 in *Nurr1*-overexpressing rat NPCs on differentiation day 7 was determined. More TH+ cells were found in FBS-treated cultures, although there was also a significant increase in the number of MAP2+ cells. (**B**) FBS has a neurogenic effect only in the presence of NURR1. The number of MAP2+ cells did not increase in the absence of *Nurr1* overexpression. FBS induced an increase in the number of GFAP+ cells in both *Nurr1* retrovirus-transduced and nontransduced cultures. An anti-GFAP antibody was used as an astrocyte-specific marker. **p < 0.01. Scale bar, 20 μm. The error bars are the S.E. from two independent experiments.

To examine whether the neurogenic effect of FBS is limited to DA neurons, we compared the levels of a general neuronal marker, microtubule associated protein 2 (MAP2), on differentiation day 7. FBS treatment significantly increased MAP2 levels, although the effect was less prominent than that on TH (Fig. 2A; control: 2.3 ± 1% [TH/DAPI], 10.6 ± 1.9% [MAP2/DAPI]; 0.01% FBS: 8.7 ± 2.7% [TH/DAPI], 21.1 ± 3.8% [MAP2/DAPI]). This suggested that, in addition to inducing the differentiation of DA neurons, FBS treatment induced neuronal differentiation in general. Furthermore, the levels of glial fibrillary acidic protein (GFAP), an astrocytic marker, increased in the presence of FBS both with and without *Nurr1* transduction, whereas MAP2 levels increased only with *Nurr1* transduction (Fig. 2B; with *Nurr1* transduction, control: 10 ± 1.7% [MAP2/DAPI], 3.2 ± 0.8% [GFAP/DAPI]; 0.01% FBS: 20.5 ± 2.9% [MAP2/DAPI], 9.6 ± 1.7% [GFAP/DAPI]; without *Nurr1* transduction, control: 3.4 ± 0.9% [MAP2/DAPI], 3.8 ± 0.9% [GFAP/DAPI]; 0.01% FBS: 4.6 ± 0.8% [MAP2/DAPI], 9.6 ± 1.4% [GFAP/DAPI]). Therefore, treatment with 0.01% FBS induced MAP2+ neurons in the presence of *Nurr1*.

### Identification of key components in FBS that promote DA neuron differentiation

Because FBS effectively induced the differentiation and maintenance of DA neurons, we hypothesized that a key component of FBS promotes DA neuron differentiation. To identify that critical component, we used a mixture of the components of FBS^12^ to treat *Nurr1*-overexpressing rat cortical NPCs.

**Figure 3:**
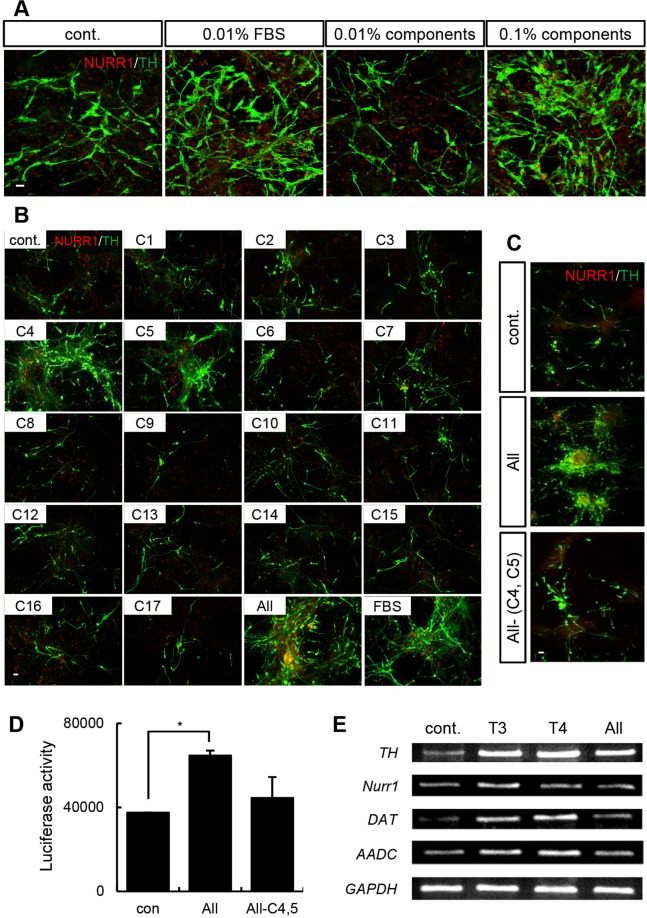
T3 and T4 are key components of FBS required for DA neuron differentiation. (**A**) Immunocytochemistry showing that treatment with the chemical mixture increased the number of NURR1+ and TH+ DA neurons. The chemicals were diluted to a concentration equivalent to that found in FBS. The 0.1% chemical mixture had an effect similar to that of 0.01% FBS in *Nurr1*-overexpressing rat NPCs. (**B**) Cells were immunostained with anti-NURR1 and anti-TH antibodies on differentiation day 7. Untreated cells were used as the negative control, and cells treated with the chemical mixture containing the 17 components of FBS acted as the positive control. There were more TH+/NURR1+ cells upon treatment with candidate factors C4 and C5 exhibited than upon treatment with the other chemicals. (**C**) Cells treated without the candidate factors (C4: T3; C5: T4) were immunostained with anti-NURR1 and anti-TH antibodies on differentiation day 7. The number of DA neurons in the cultures decreased dramatically when C4 and C5 were eliminated. (**D**) A TH promoter assay in *Nurr1-Mash1*-overexpressing rat NPCs treated with the chemical mixture from which C4 and C5 were eliminated. (**E**) RNA was extracted from cells that were treated with T3 (50 pg/ml) or T4 (5 pg/ml) for 10 days. RT-PCR analysis was performed to examine the expression of DA neuron–related genes (*Th*, *Nurr1*, *Dat*, and *Aadc*). *GAPDH* expression was used as a loading control. The error bars are the S.E. *p < 0.05. Scale bar, 20 μm. The experiment was repeated twice with similar results.

The name and concentration of each component are listed in Supplementary Table 1. In agreement with our hypothesis, the number of DA neurons significantly increased following treatment with the component mixture to an extent similar to that observed with FBS treatment (Fig. 3A), indicating that the mixture contained a critical component for the promotion of the differentiation of DA neurons. To identify that key component, NPCs overexpressing both *Nurr1* and *Mash1* were treated with the individual components of FBS during the differentiation period. We treated rat cortical NPCs with a *Nurr1-Mash1* retrovirus to induce rapid DA neurogenesis because MASH1 in cooperation with NURR1 accelerates functional DA neuronal differentiation^20^. Immunostaining suggested that only the 4th component (C4) and the 5th component (C5) increased the number of TH+ neurons (Fig. 3B). To further clarify their involvement in this process, C4 and C5 were removed from the component mixture, and this resulted in a significant decrease in the number of DA neurons (Fig. 3C). To investigate whether C4 and C5 affect TH expression at the transcriptional level, we performed a TH promoter assay. Similar to the results observed with immunostaining, TH promoter activity was reduced after eliminating C4 and C5 from the mixture (Fig. 3D; luciferase activity, control: 37,421 ± 123.6; complete mixture: 64,620 ± 2397.6; mixture without C4 and C5: 44,424 ± 9986.8). Notably, C4 and C5 are the thyroid hormones T3 and T4. As shown in Fig. 3B, treatment with T3 or T4 alone was sufficient to generate TH-immunopositive cells from rat cortical NPCs. To determine the efficacy of thyroid hormones in promoting DA neuron differentiation, we added various concentrations of the hormones to rat cortical NPC cultures in which *Nurr1* and *Mash1* were overexpressed to stimulate DA neuron differentiation. The number of NURR1+ and TH+ neurons gradually increased in a concentration-dependent manner upon treatment with up to 50 pg/mL T3 (Supplementary Fig. 2A). TH promoter activity also increased steadily in the presence of up to 50 pg/mL T3 (Supplementary Fig. 2B). Using RT-PCR, we observed that the expression genes associated with the DA phenotype, such as *TH*, *Nurr1*, DA transporter (*Dat*), and aromatic L-amino acid decarboxylase (*Aadc*), was increased by the thyroid hormones (Fig. 3E). Furthermore, when T3 and T4 were applied to rat E14 VM NPCs endogenously expressing NURR1, the number of TH+ cells increased in a concentration-dependent manner (Supplementary Fig. 2C; TH/DAPI, control: 1.65 ± 0.5%; T3 1.5 pg/mL: 2.46 ± 0.6%; T3 50 pg/mL: 3.44 ± 0.8%; T4 0.15 pg/mL: 2.81 ± 0.7%; T4 5 pg/mL: 4.63 ± 0.8%). Taken together, these findings provide evidence that T3 and T4 have a concentration-dependent effect on the differentiation of DA neurons in NPC cultures.

### Synthesized T3 and T4 derivatives are selectively effective for DA neuron induction

**Figure 4:**
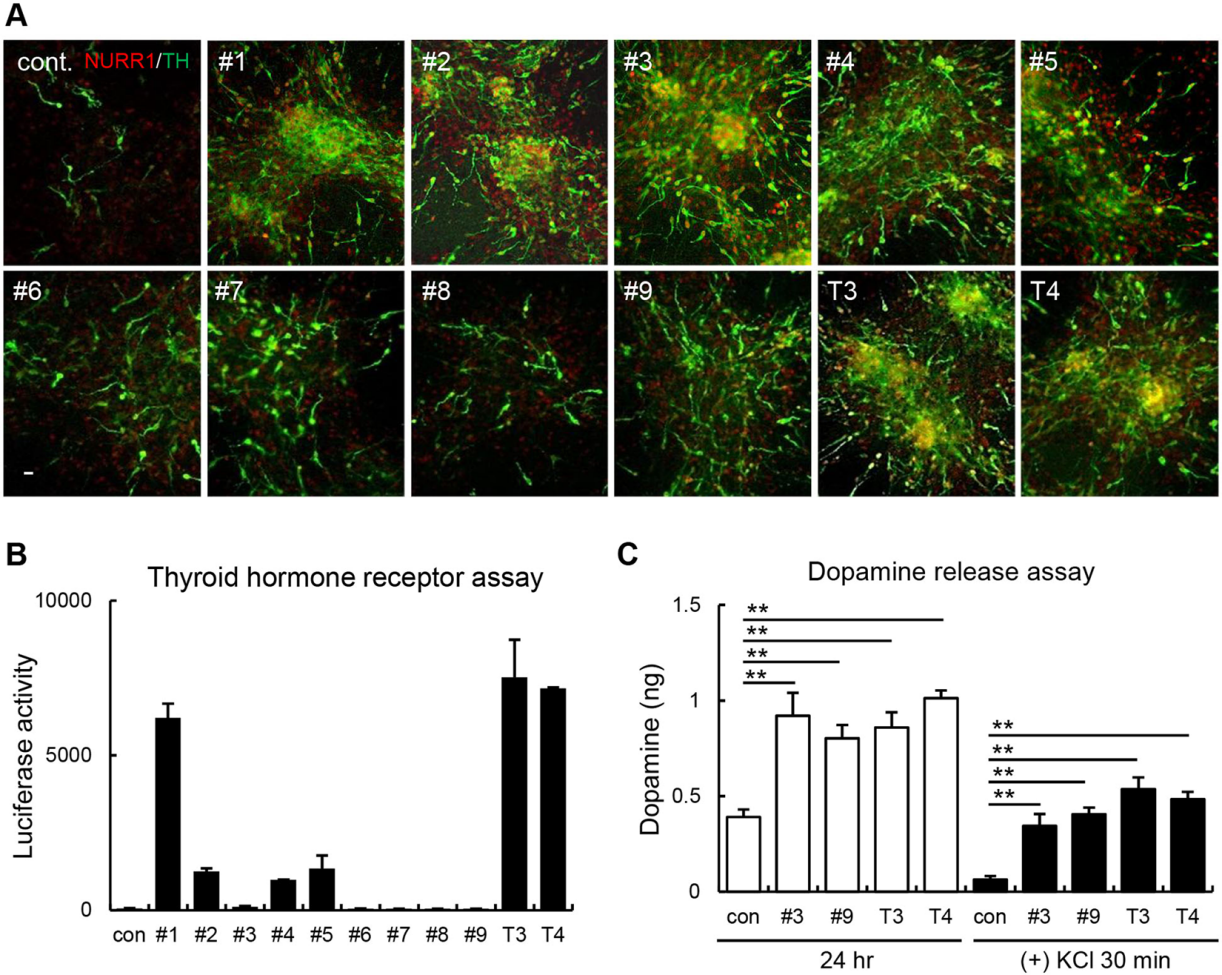
Derivatives #3 and #9 effectively induced DA neurons. (**A**) *Nurr1-Mash1*-overexpressing rat cortical NPCs were treated with thyroid hormone derivatives and differentiated for 7 days. DA neurons were immunostained with NURR1 and TH antibodies. Cells treated with T3 and T4 were used as positive controls. (**B**) A luciferase assay was performed to measure the affinity of the derivatives for TRα. Derivatives #3, #6, #7, #8, and #9 showed a low affinity for TRα. (**C**) The amount of DA released from presynaptic DA neurons was detected to determine the functional dopaminergic activity of the rat *Nurr1-Mash1*-overexpressing NPCs on differentiation day 7. DA release was induced by stimulation with 56 mM KCl. **p < 0.01. Scale bar, 20 μm. The error bars are the S.E. from three independent experiments.

Elevations in thyroid hormone levels beyond the normal range can cause unintentional weight loss, sleep disruption, and Grave’s disease and can even cause death if the levels are severely affected^21–24^. Thyroid hormones play important roles in DA neuron differentiation and maintenance^18^, but high concentrations of these hormones can cause hyperthyroidism. Therefore, the development of thyroid hormone derivatives that have similar effects on DA neurons without the associated effects of hyperthyroidism is important to generate clinical agents for treating PD. We thus synthesized a series of T3 and T4 derivatives that have weak affinity for the thyroid hormone receptor but are still effective for DA neuron differentiation (Supplementary Fig. 3). We treated *Nurr1-Mash1*-overexpressing rat cortical NPCs with nine different derivatives to determine their efficacy for DA neuron induction. We observed that, compared with control, derivatives #1, #2, #3, #4, #5, and #9 promoted a significant increase in the number of NURR1+ and TH+ neurons (Fig. 4A). To identify the derivatives that partially activate the thyroid hormone receptor and fully induce DA neurogenesis, we tested the activity of each derivative on TRα using a luciferase assay system. Among the derivatives, derivatives #3 and #9 appeared to have weak activity on TRα (based on the luciferase assay) but strong effects on DA neuron induction (Fig. 4B; luciferase activity, control: 55 ± 10.1; #3: 105 ± 31.3; #9: 43 ± 6.4; T3: 7,522 ± 1211.5; T4: 7,164 ± 31.1). The number of TH+ neurons induced by treatment with derivative #3 was higher than that induced by derivative #9. Thus, derivatives #3 and #9 possess high DA neuron-inducing effects and seem to be weak agonists for the thyroid hormone receptor. To characterize them further, we performed RNA-seq analysis to determine the changes in the gene expression profiles induced by the thyroid hormones and their derivatives in *Nurr1-Mash1*-overexpressing NPCs. DA neuron-related gene expression is presented using a heat map (Supplementary Fig. 4). The first and second lanes show the results pertaining to nontransduced NPCs and untreated *Nurr1-Mash1*-overexpressing NPCs (negative control), respectively. On the whole, the chemically treated groups (T3, T4, #3, and #9) showed similar patterns of expression, but compared with the cells treated with thyroid hormones, the cells treated with derivatives #3 and #9 showed lower expression levels of several glial cell-related genes, such as oligodendrocyte transcription factor 3 (*Olig3*), *Nkx6*.*1*, and *Gli2*. In contrast, treatment with the derivatives, relative to treatment with thyroid hormones, upregulated the expression of several neurotrophic genes (*L1cam*, *Wnt5a*, and *Tbc1d24*). To further elucidate the function of the derivatives and thyroid hormones in DA neuron induction, we performed a DA release assay. We used KCl stimulation for 30 min to measure DA release induced by membrane depolarization. Treatment with both the derivatives and thyroid hormones induced more DA release than no treatment both before and after KCl stimulation (Fig. 4C; released DA, 24 h, control: 0.4 ± 0.1 ng; #3: 0.9 ± 0.1 ng; #9: 0.8 ± 0.1 ng; T3: 0.9 ± 0.2 ng; T4: 1 ± 0.1 ng; KCl 30 min, control: 0.1 ± 0 ng; #3: 0.3 ± 0.1 ng; #9: 0.4 ± 0.1 ng; T3: 0.5 ± 0.1 ng; T4: 0.5 ± 0.1 ng). Collectively, these results show that the thyroid hormone derivatives induced functional DA neurons and had weak activity on TRα, which indicates that they may have therapeutic potential for the treatment of PD with reduced adverse effects.

### DA-related neurological effects of thyroid hormones and their derivatives on human NPCs

**Figure 5:**
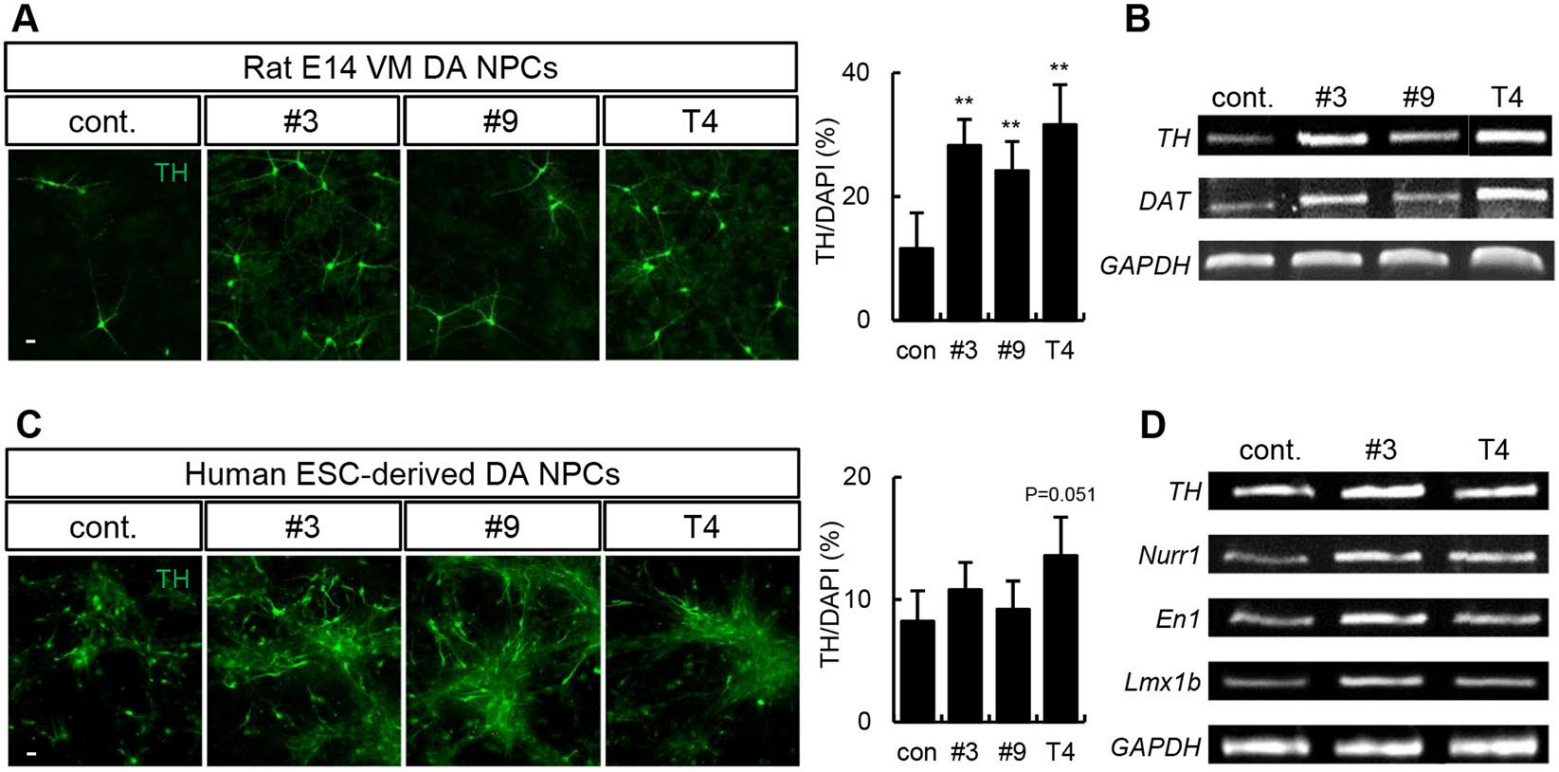
Thyroid hormones and their derivatives induce DA neurons in rat and human NPCs endogenously expressing NURR1. (**A**) Rat VM NPCs treated with derivatives #3 or #9 or T4 differentiated into DA neurons after 10 days. The cells were immunostained with an anti-TH antibody. TH expression increased following treatment. (**B**) RT-PCR analysis of DA neuron-related genes (*TH* and *Dat*) in derivative #3-, derivative #9-, and T4-treated cells. (**C**) Human ESC-derived DA NPCs were treated with derivative #3, derivative #9, or T4 during the differentiation period. On differentiation day 14, immunocytochemistry was performed using an anti-TH antibody. (**D**) The mRNA levels of DA neuron–related genes (*TH*, *Nurr1*, *En1*, and *Lmx1b*) increased following treatment with derivative #3 and T4. *GAPDH* expression was used as a loading control. **p < 0.01, Scale bar, 20 μm. The error bars are the S.E. from two independent experiments.

To further assess the physiological role of thyroid hormone derivatives in the differentiation of NPCs into DA neurons, dopaminergic potential was measured in rat VM NPCs. Cells were treated with derivatives #3 and #9 for 10 days during the differentiation period. The derivatives significantly increased the number of TH+ neurons (Fig. 5A; control: 11.6 ± 5.8%; #3: 28.2 ± 4.3%; #9: 24.2 ± 4.7%; T4: 31.6 ± 6.5%) and the mRNA levels of DA neuron–related genes compared with the control group (Fig. 5B).

To examine the translatability of those effects to human cells, we tested the potential of the derivatives to induce DA neurons in human NPC cultures. Human embryonic stem cell (ESC)-derived NPCs were differentiated into DA neurons for 2 weeks using derivative #3, derivative #9, or thyroid hormone. The number of TH+ neurons increased slightly in human NPC cultures following treatment with thyroid hormones or their derivatives (Fig. 5C; control: 8.2 ± 2.5%; #3: 10.8 ± 2.2%; #9: 9.2 ± 2.3%; T4: 13.6 ± 3.2%). DA neuron-related genes such as engrailed-1 (*En1*) and LIM homeobox transcription factor 1b (*Lmx1b*) were also increased by the treatment (Fig. 5D). We evaluated the expression of other types of neural lineage cell markers in human NPCs upon treatment with the derivatives. The derivatives did not affect astrocytes or nondopaminergic neurons, such as GABAergic neurons (Supplementary Fig. 5A). These results suggest that thyroid hormones and their derivatives are effective in both human and rat DA neurons.

### Efficacy of thyroid hormones and their derivatives for protecting DA neurons from neurotoxic damage

**Figure 6:**
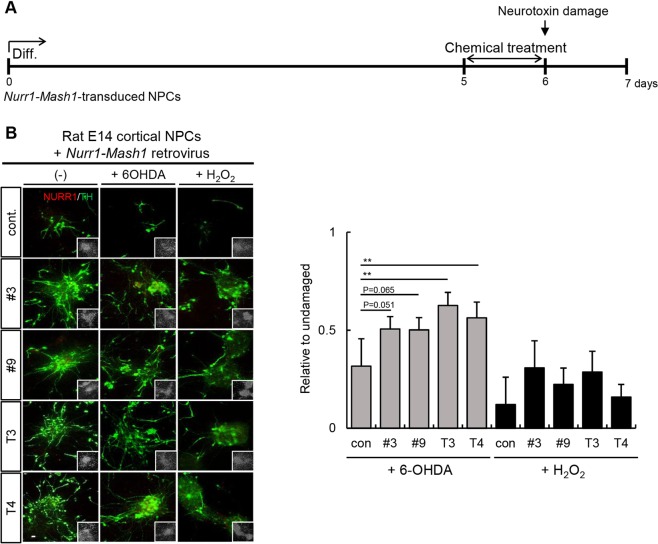
Thyroid hormones and their derivatives can protect DA neurons from neurotoxic damage. (**A**) Timeline of the experimental protocol for the dopaminergic neuron protection test. (**B**) *Nurr1-Mash1*-overexpressing rat NPCs were treated with thyroid hormones or their derivatives for 24 h on differentiation day 5. The following day, a neurotoxic insult (6-OHDA for 18 h or H_2_O_2_ for 3 h) was delivered to the DA neurons. On differentiation day 7, DA neurons were immunostained with an anti-TH antibody. TH+ neurons were detected following treatment with both the thyroid hormones and their derivatives. **p < 0.01. Scale bar, 20 μm. The error bars are the S.E. from two independent experiments.

**Figure 7:**
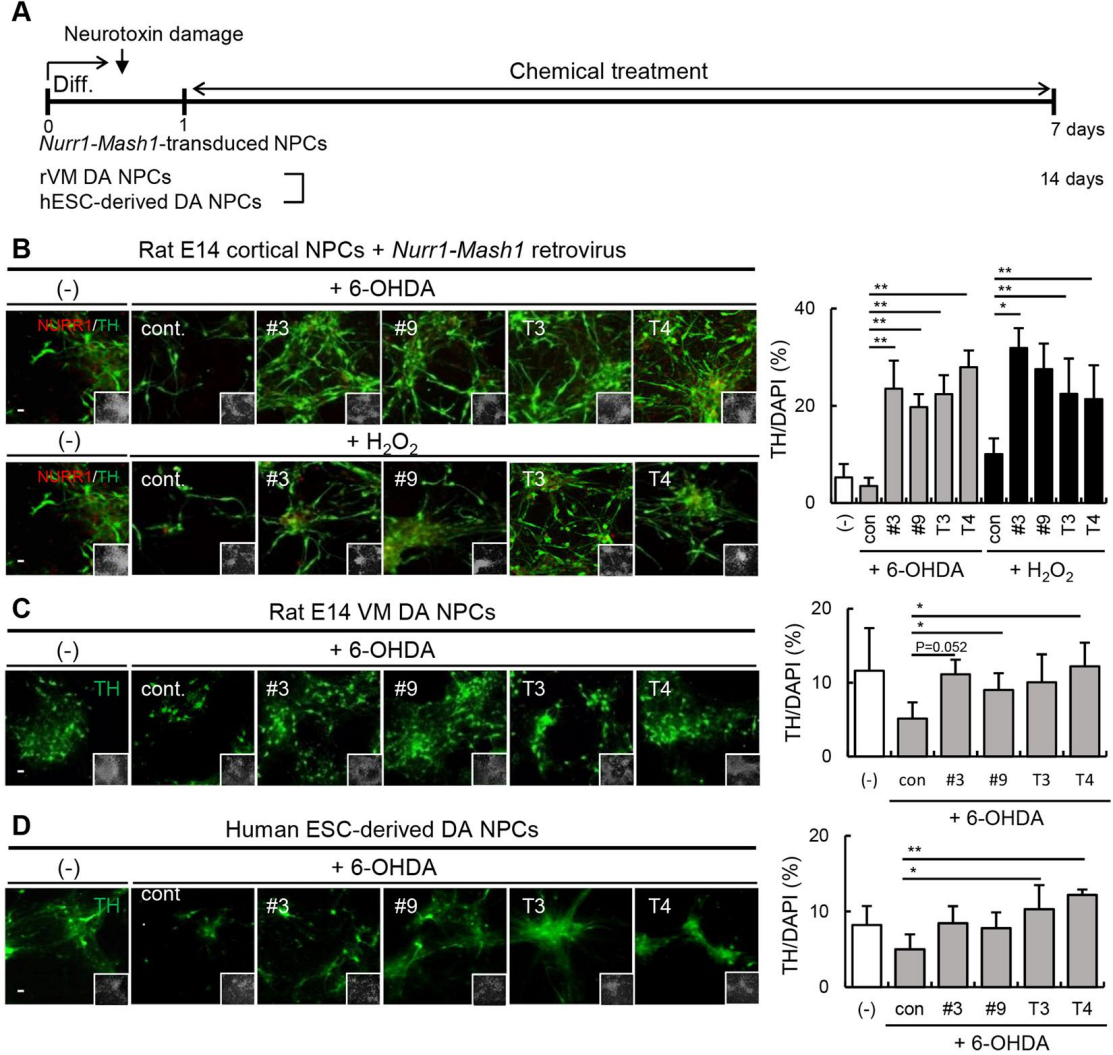
Thyroid hormones and their derivatives can restore DA neurons from neurotoxic damage. (**A**) Timeline of the experimental protocol of the dopaminergic neuron recovery test. (**B**) After a neurotoxic insult (6-OHDA for 18 h or H_2_O_2_ for 3 h) was administered to *Nurr1-Mash1-*overexpressing rat NPCs, cells were treated with thyroid hormones or their derivatives throughout the differentiation period. Under conditions of neurotoxic damage, the thyroid hormones and their derivatives significantly increased the number of TH+ neurons. (**C**) Rat VM NPCs and (**D**) human ESC-derived NPCs were incubated with 6-OHDA (18 h) and subsequently incubated with the thyroid hormone derivatives for 14 days after differentiation. The number of TH+ neurons increased following treatment with the thyroid hormone derivatives. *p < 0.05, **p < 0.01. Scale bar, 20 μm. The error bars are the S.E. from two independent experiments.

To investigate the clinical applicability of thyroid hormone derivatives, we analyzed their ability to protect DA neurons from neurotoxin exposure. We used 6-hydroxydopamine (6-OHDA) and H_2_O_2_ to induce DA neuronal damage. 6-OHDA is a specific DA neurological toxin, and H_2_O_2_ induces apoptosis of neuronal cells^25,26^. *Nurr1-Mash1-*overexpressing rat DA neuronal cells differentiated for 5 days were treated with thyroid hormones or their derivatives for 24 h and then exposed to 6-OHDA (20 μM, 18 h) or H_2_O_2_ (100 μM, 3 h) (Fig. 6A). Compared to the untreated control cultures, the cultures treated with thyroid hormones or their derivatives for one day exhibited more TH+ neurons, implying that the thyroid hormones and their derivatives had protective effects (Fig. 6B; 6-OHDA, control: 0.3 ± 0.1%; #3: 0.5 ± 0.1%; #9: 0.5 ± 0.1%; T3: 0.6 ± 0.2%; T4: 0.6 ± 0.1%; H_2_O_2_, control: 0.1 ± 0.1%; #3: 0.3 ± 0.1%; #9: 0.2 ± 0.1%; T3: 0.3 ± 0.1%; T4: 0.2 ± 0.1%). Next, we determined whether the derivatives can promote the recovery of DA neurons following neurotoxin exposure. We induced cellular damage in *Nurr1-Mash1*-overexpressing rat cortical NPCs with 6-OHDA (20 μM, 18 h) or H_2_O_2_ (100 μM, 3 h). Toxin-pretreated NPCs were then treated with a thyroid hormone or derivative for 1 week, and the number of TH+ neurons remaining was quantified (Fig. 7A). Treatment with both the thyroid hormones and their derivatives increased recovery after exposure to both neurotoxins (Fig. 7B; TH/DAPI, control: 5.3 ± 2.8%, 6-OHDA; control: 3.5 ± 1.7%; #3: 23.5 ± 5.8%; #9: 19.7 ± 2.6%; T3: 22.4 ± 3.9%; T4: 27.9 ± 3.5%; H_2_O_2_, control: 10 ± 3.3%; #3: 31.9 ± 4.1%; #9: 27.5 ± 5.3%; T3: 22.4 ± 7.3%; T4: 21.4 ± 7%). Moreover, treatment with thyroid hormones and their derivatives appeared to rescue DA neurons from damage in both rat VM NPCs and human NPCs endogenously expressing NURR1 (Fig. 7C; TH/DAPI, control: 11.6 ± 5.8%; 6-OHDA, control: 5.1 ± 2.2%; #3: 11.1 ± 2%; #9: 9 ± 2.3%; T3: 10 ± 3.8%; T4: 12.2 ± 3.2%; Fig. 7D; TH/DAPI, control: 8.2 ± 2.5%; 6-OHDA, control: 5 ± 2%; #3: 8.5 ± 2.2%; #9: 7.8 ± 2.1%; T3: 10.3 ± 3.2%; T4: 12.2 ± 0.7%). In addition, we evaluated changes to other neurons induced by 6-OHDA exposure and treatment with the derivatives in rat VM DA NPCs. However, 6-OHDA exposure did not change the number of MAP2+ cells. Treatment with the derivatives increased the expression of MAP2 slightly through restoring DA neurons (Supplementary Fig. 5B). These results suggest that thyroid hormones and their derivatives are effective at protecting DA neuronal function and promoting recovery following damage.

## Discussion

FBS is widely used as a growth factor in cell culture media, and it contains many hormones and chemical compounds^12,13^. However, FBS induces an astrocytic fate in NPC cultures and can interfere with the differentiation of cells toward a neuronal fate^14^. Thus, using FBS in NPC cultures for the induction of pure neuronal cultures is not recommended; instead, the use of serum-free medium containing growth factors such as basic fibroblast growth factor (bFGF) and epidermal growth factor is recommended^27^. The functional relationship between FBS and DA neurogenesis has yet to be fully elucidated.

In this study, we examined the effects of FBS on DA neuron induction using *Nurr1*-expressing NPCs. FBS (0.01%) successfully increased *Nurr1* expression and the number of TH+ neurons *in vitro*. Compared with untreated cells, FBS-treated cells showed a more mature morphology, including longer and more elaborate neurites indicative of more complex synaptic function^28–30^. Under normal conditions, when *Nurr1* was transduced into NPCs to generate DA neurons, the highest proportion of DA neurons was observed 2 to 3 days after differentiation^19^. Monitoring DA neuron differentiation revealed that the effect of FBS became more prominent with the passage of time (Fig. 1). Therefore, our results suggest that the chemical components of FBS may have an important role in the generation of DA neurons.

FBS induced the differentiation of both DA neurons and other neurons expressing *Nurr1*. Interestingly, when cortical NPCs that were not transduced with the *Nurr1* retrovirus were treated with FBS, astrocytic differentiation increased, but there was no effect on neurons (Fig. 2). These results suggest that the neurogenic effect of FBS is NURR1-dependent.

Next, we tested the chemical components of FBS to identify the component that promotes the induction of TH+ neurons. We purchased candidate chemical components present in FBS and diluted them appropriately to generate a chemical solution that mimicked FBS^12^. As a result, we observed that T3 and T4 affected the induction of DA neurons. The addition of T3 or T4 to the differentiation medium increased the number of DA neurons and the mRNA levels of DA neuron-related genes in the cell cultures (Fig. 3). Thyroid hormones have been implicated in neuronal migration, differentiation, and oligodendrocyte myelination^31^. For instance, T3 blocks proliferation and induces the differentiation and maturation of oligodendrocyte progenitor cells into oligodendrocytes via the regulation of myelin basic protein^32–34^. We observed that T3 and T4 assisted in the maintenance of DA neurons, a phenomenon that involved increased *Nurr1* expression. Several other hormones are present in FBS; however, no other component tested in our study showed any effect on the induction of DA neurons.

Hypothyroidism is known to decrease the number of DA neurons. Mice with a genetic mutation that causes hypothyroidism show a loss of midbrain DA neurons and display spontaneous and persistent unilateral circling behavior^16^. However, the precise relationship between thyroid hormones and DA neurons is unknown. A previous study reported that thyroid hormones assist in the regeneration of DA neurons by enhancing *Otx2* expression through TRα1 and activating *Nurr1* and neurogenin 2 (*Ngn2*) expression through *Otx2*^18^. However, the authors did not show whether thyroid hormone receptors bind to and regulate *Otx2* directly. In addition, even though thyroid hormones are effective at inducing the differentiation of DA neurons, continuous stimulation by thyroid hormones can lead to pathological dysfunction and cause complications in many organs. Commonly reported symptoms of high thyroid levels include disturbed sleep, weight loss, tremors, anxiety, and tiredness, and these symptoms can be accompanied by other symptoms, such as bulging or dry eyes (diplopia), also known as Grave’s ophthalmopathy. A lack of timely medical intervention for this illness can lead to severe side effects or death^21–24^.

Therefore, we designed and tested derivatives of thyroid hormones in this study. We used a luciferase assay-based reporter system to measure the activities of thyroid hormone receptor derivatives and selected the derivatives that were effective but had low activity on the receptor. In that way, we determined that derivatives #3 and #9 had lower activity on TRα than T3 and T4 while maintaining their effects on DA neurons. Derivatives #1, #2, #4, and #5 were also effective at inducing DA neurons; however, they did not show low activity on the TRα receptor (Fig. 4). The DA-inducing effects were observed in both rat and human NPCs (Fig. 5). Moreover, the derivatives offered protection against the neurotoxins 6-OHDA and H_2_O_2_. Pretreatment with the derivatives for 1 day prior to neurotoxic damage attenuated impairments in DA neurogenesis (Fig. 6). When the neurotoxic insults were administered in a different order to test the effect of the treatments on recovery, the thyroid hormones and their derivatives successfully promoted the regeneration of DA neurons following neurotoxic damage (Fig. 7). This suggests that the administration of thyroid hormone derivatives may provide therapeutic benefits to PD patients.

We did not elucidate the mechanism by which the derivatives with low affinity for TRα induced the differentiation of DA neurons. We hypothesize that thyroid hormones and their derivatives might not bind solely to TR but might also act upon other currently unidentified receptors. In contrast to thyroid hormones, the derivatives do not preferentially bind to TR, possibly for structural reasons.

The use of these derivatives for the treatment of PD has certain limitations. The administration of the derivatives for prolonged periods or at high concentrations may overstimulate the thyroid gland. Although the derivatives have a lower affinity for the thyroid receptor than T3 and T4, they can still bind to the receptor, albeit weakly. Therefore, we are currently developing effective derivatives that are more suitable for PD treatment. Whether thyroid hormones elevate the expression of *Nurr1* or block the degradation of the NURR1 protein remains unclear. Therefore, further investigations are required to determine the mechanism by which DA neurogenesis occurs in the presence of thyroid hormones.

In conclusion, we demonstrated that low-dose FBS induces DA neuron differentiation, partly through the action of the thyroid hormones present in FBS. Furthermore, we have developed effective derivatives of thyroid hormones that may become novel therapeutic candidates for the treatment of PD. Our observations are relevant for PD therapy and represent a step forward in the development of new medications for PD.

## Materials and Methods

### Approval of animal experiments

The protocols for the use of animals in these studies were approved by the Institutional Animal Care and Use Committee of Hanyang University (approval number: Hanyang University IACUC-2016-0194A), and all experiments were carried out in accordance with the approved protocols.

### Primary rat NPC cultures

After removing the embryos from female Sprague-Dawley (SD) rats (DaeHan BioLink, Seoul, Korea) at embryonic day 14 (E14), the cerebral cortex was dissected, and single cells were isolated from the tissue. NPCs were seeded on plates coated with poly-L-ornithine (PLO; 15 μg/mL, Sigma-Aldrich, St Louis, MO, USA)/fibronectin (FN; 1 μg/mL, Sigma-Aldrich). The growth medium was composed of serum-free medium (N-2) containing 20 ng/mL bFGF (R&D Systems, Minneapolis, MN, USA). Confluent cells were incubated for 1 h with Ca^2+^/Mg^2+^-free Hank’s balanced salt solution (Invitrogen, Carlsbad, CA, USA) for single cell dissociation. After dissociation, the cells were plated on PLO/FN-coated glass slides in 24-well or 6-well plates. For neuronal differentiation, bFGF was removed from the medium, and 0.2 mM ascorbic acid (Sigma-Aldrich) was added.

### Primary rat VM NPC cultures

The VM was dissected from female SD rat embryos at E14. Single cells were isolated from the tissue and plated on PLO/FN-coated glass slides in 24-well plates. Cells were cultured in N-2 growth medium containing 20 ng/mL bFGF. The differentiation medium consisted of N-2 supplemented with 0.2 mM ascorbic acid and 250 μg/mL dibutyryl cyclic AMP (db-cAMP; Sigma-Aldrich).

### Human NPC cultures

The human ESC culture protocol (HYE-08-03) was approved by the Hanyang University Institutional Review Board. Human NPCs were derived from the human ESC line H9 (University of Wisconsin, Madison, WI, http://www.wisc.edu; CHA13, CHA Stem Cell Institute, http://www.cha.ac.kr) using an *in vitro* differentiation protocol involving neural induction on γ-irradiated sonic hedgehog-overexpressing MS5 stromal cells followed by several cell passages to select for NPCs, as previously described^35^. Human NPCs were cultured in serum-free insulin transferrin-selenium medium^36^ containing 20 ng/mL bFGF. For neuronal differentiation, bFGF was removed from the medium, and 0.2 mM ascorbic acid and 250 μg/mL db-cAMP were added.

### Retrovirus production and transduction

For dopaminergic neuronal differentiation in NPCs, *Nurr1*- or *Nurr1-Mash1*-expressing retroviruses were produced from 293GPG packaging cells. DNA constructs were constructed using a pCL retroviral vector plasmid. The transfection of 293GPG cells with DNA was performed using Lipofectamine 2000 (Invitrogen). The supernatant containing viral particles was collected 72 h after transfection. The viral supernatant was added to primary NPCs for 2 h in the presence of polybrene (2 μg/mL, Sigma-Aldrich).

### Chemical treatment

NPCs were treated with FBS (Invitrogen), NGS (Invitrogen), serum replacement (SR; Invitrogen), and various chemical components of FBS^12^ (Sigma-Aldrich) during dopaminergic differentiation. The derivatives of the thyroid hormones were provided by the Korea Research Institute of Chemical Technology, and they were dissolved in dimethyl sulfoxide (DMSO). These derivatives were mixed with medium immediately prior to treatment.

### Synthesis of thyroid hormone derivatives

The synthesis of levothyroxine T4 derivatives #4, #7, and #9 is depicted in Supplementary Fig. 3. Commercially available levothyroxine T4 was reacted with 2,2-dimethoxypropane in the presence of hydrochloric acid to obtain levothyroxine methyl ester #1, which was subsequently reacted with acetic anhydride in the presence of pyridine to produce acetyl-protected compound #3 and then hydrolyzed to produce carboxylic acid #4. Amine hydrochloride #1 underwent Boc protection followed by hydrolysis to produce carboxylic acid #5. The condensation of carboxylic acid #5 with appropriate amines followed by acidic deprotection was used to generate amine hydrochlorides #7 and #9 (details are in the Supplementary Materials and Methods).

### Reverse transcriptase-polymerase chain reaction (RT-PCR)

Total RNA was extracted using TRI reagent (Molecular Research Center Inc., Cincinnati, OH, USA). The cDNA was synthesized using a Superscript kit (Invitrogen) in a final volume of 20 μL, and PCR was performed using the primers and conditions shown in Supplementary Table 2.

### Immunocytochemistry

Cells were fixed with 4% paraformaldehyde (Sigma-Aldrich). After 15–20 min, the cells were washed three times in 0.1% bovine serum albumin/phosphate-buffered saline wash buffer and blocked for 1 h using 0.3% Triton X-100 (Sigma-Aldrich) and 10% NGS (Invitrogen). After blocking, the cells were incubated with primary antibodies overnight at 4 °C. The cells were incubated with biotin for 30 min and then with a fluorescence (DTAF, rhodamine, or Cy3)-tagged antibody; Jackson Immunoresearch Laboratories, West Grove, PA, USA) for 1 h. The primary antibodies were as follows: anti-TH antibody (1:2000; Pel-Freez, Rogers, AR, USA), anti-NURR1 antibody (1:2000; Perseus Proteomics, Meguro, Tokyo, Japan), anti-MAP2 antibody (1:1000; Sigma-Aldrich), anti-GFAP antibody (1:2000; DAKO, Glostrup, Denmark), and anti-GABA antibody (1:5000; Sigma-Aldrich). The immunostained cells were mounted using Vectashield mounting medium with 4′,6-diamidino-2-phenylindole (DAPI) (Vector Laboratories, Burlingame, CA, USA). They were visualized and photographed using an epifluorescence microscope (Leica Microsystem, Wetzler, Germany).

### Promoter assay

Thyroid hormone receptor binding activity was measured using a GAL4 system, which comprised a plasmid expressing the fusion protein of the ligand-binding domain of TRα and the DNA-binding domain of GAL4. To estimate TH promoter activity, we inserted a 6.0-kb fragment of the rat TH promoter upstream of the luciferase gene obtained from the pGL3-basic plasmid^37^. A pRSV-Renilla-luciferase plasmid expressing Renilla luciferase was used for normalization. On differentiation day 6, the cells were transfected with plasmids using Lipofectamine 2000. The following day, the cells were harvested and lysed for 10 min using cell lysis buffer (BD Pharmingen, San Diego, CA). The cell lysates were mixed with Renilla luciferase reagent (Enhanced Luciferase Assay kit, BD Pharmingen). Luciferase activity was measured using a luminometer (Berthold Detection Systems, Huntsville, AL, USA).

### Dopamine release assay

DA release was detected using a dopamine enzyme-linked immunosorbent assay (ELISA) kit (Labor Diagnostika Nord, Nord-horn, Germany) according to the manufacturer’s instructions. To determine the amount of DA released from differentiated cells with or without *Nurr1* and *Mash1* transduction, differentiation medium was collected from the cells 48 h after a medium change. DA release induced by depolarization was also measured in the differentiated cells. Cells at the terminal differentiation stage on day 7 were incubated in differentiation medium with (induced release) or without (basal release) 56 mM KCl for 30 min, and the medium was subsequently collected to measure DA. The collected medium was stabilized with 4 mM sodium metabisulfite containing 1 mM EDTA. DA levels were calculated from the standard curve plotted using the absorbance readings (linear scale, y-axis) of serially diluted concentrations of DA (logarithmic scale, x-axis).

### Neurotoxin treatment

6-Hydroxydopamine (6-OHDA, 20 μM, 18 h; Sigma-Aldrich) and hydrogen peroxide (H_2_O_2_, 100 μM, 3 h; Sigma-Aldrich) were used to induce DA neuronal damage. To determine the protective effects of the thyroid hormones and their derivatives, a neurotoxic insult was introduced to in differentiated DA neurons using 6-OHDA or H_2_O_2_ on differentiation day 6. Following the delivery of the insult, the cells were washed twice in medium, followed by incubation in fresh medium. To chart the recovery of the cells, a neurotoxic insult was delivered to rat cortical NSCs on differentiation day 0. After treatment, the cells were washed twice in medium and then cultured for 1 week in fresh differentiation medium containing thyroid hormones or their derivatives.

### Cell counting and statistical analysis

Cell counting was performed manually using a uniform random selection of five to ten microscopic fields per well with three to four wells per experimental condition. All experiments were performed at least twice to show statistical significance. The data are expressed as the means ± standard error (S.E.). Statistical analysis was performed using OriginPro 8 software (OriginLab, Northampton, MA). One-way analysis of variance was used when there were more than two groups. Tukey test was used for all posttests because we had the same sample size in each experimental group. When two groups were compared, paired t test was performed with SigmaPlot for Windows, version 10.0 (Systat Software GmbH, Erkrath, Germany).

## Supplementary information

Supplementary Information

## Data Availability

The raw/processed data required to reproduce these findings cannot be shared at this time because they are part of an ongoing study.
