# Supplementary material for: Dopamine neuron induction and the neuroprotective effects of thyroid hormone derivatives
Source: Sci Rep. 2019 Sep 20;9:13659. doi: 10.1038/s41598-019-49876-6 (PMC6754465; doi:10.1038/s41598-019-49876-6)
Supplement: Supplementary file 1 — Supplementary Information [file 41598_2019_49876_MOESM1_ESM.docx]

**SUPPLEMENTARY MATERIALS AND METHODS**

**Dopamine neuron induction and the neuroprotective effects of thyroid hormone derivatives**

Eun-Hye Lee,^1^ Sang-Mi Kim,^1^ Chun-Hyung Kim,^2^ Suvarna H. Pagire,^3^ Haushabhau S. Pagire,^3^ Hee Yong Chung,^1,4,5,*^ Jin Hee Ahn,^3,*^ and Chang-Hwan Park^1,4,5,*^

^1^Hanyang Biomedical Research Institute, Hanyang University, Seoul 04763, ^2^Paean Biotechnology, Inc. Daejeon 34028, ^3^Department of Chemistry, Gwangju Institute of Science and Technology, Gwangju 61005, ^4^Department of Microbiology, College of Medicine, and ^5^Graduate School of Biomedical Science and Engineering, Hanyang University, Seoul 04763, Korea

**Synthesis of thyroid hormone derivatives**

**General Information**

All commercial chemicals and solvents were reagent-grade and used without further purification. Products from all reactions were purified by flash column chromatography using silica gel 60 (230-400 mesh Kieselgel 60) or crystallization. ^1^H-NMR spectra were obtained on an FT-NMR Varian Gemini-300FT, Bruker AVANCE-300, or JEOL Delta-400 spectrometer with TMS as the internal reference (Supplementary Fig. 3).

**Methyl (S)-2-amino-3-(4-(4-hydroxy-3,5-diiodophenoxy)-3,5-diiodophenyl)propanoate hydrochloride (#1)**

Hydrochloric acid (1.3 mL) was added to a solution of levothyroxine T4 (1.0 g, 1.287 mmol) in 2,2-dimethoxypropane (20 mL). The reaction mixture was stirred for 12 h at 25°C. The resulting mixture was evaporated with methanol (5 mL) under reduced pressure. The precipitate was collected by vacuum filtration and washed with methanol (5 mL) to yield compound #**1** as a white solid (928 mg, 87%).

**Methyl (S)-2-((tert-butoxycarbonyl)amino)-3-(4-(4-hydroxy-3,5-diiodophenoxy)-3,5-diiodophenyl)propanoate (#2)**

A solution of di-tert-butyl dicarbonate (41.39 mg, 0.190 mmol) in dichloromethane (5 mL) was added dropwise to a solution of methyl (S)-2-amino-3-(4-(4-hydroxy-3,5-diiodophenoxy)-3,5-diiodophenyl)propanoate hydrochloride 1 (100 mg, 0.126 mmol) and Hünig's base (24.51 mg, 0.190 mmol) in dichloromethane (10 mL) and stirred at room temperature for 12 h. The reaction mixture was diluted with brine and extracted with dichloromethane. The combined organic layer was dried over anhydrous sodium sulfate and evaporated under reduced pressure to yield the crude product, which was purified by silica gel column chromatography to yield methyl (S)-2-((tert-butoxycarbonyl)amino)-3-(4-(4-hydroxy-3,5-diiodophenoxy)-3,5-diiodophenyl)propanoate #**2** (100 mg, 89%).

**Methyl (S)-2-acetamido-3-(4-(4-acetoxy-3,5-diiodophenoxy)-3,5-diiodophenyl)propanoate (#3)**

Methyl (S)-2-amino-3-(4-(4-hydroxy-3,5-diiodophenoxy)-3,5-diiodophenyl)propanoate hydrochloride 1 (20 mg, 0.024 mmol) was dissolved in dichloromethane (20 mL). Pyridine (9.56 mg. 0.121 mmol) and acetic anhydride (12.34 mg, 0.121 mmol) were added to the solution at 0°C. The mixture was stirred for 8 h at room temperature. The reaction mixture was diluted with ethyl acetate and washed with H_2_O. The organic layer was dried over MgSO_4_ and concentrated *in vacuo* to yield the desired product, methyl (S)-2-acetamido-3-(4-(4-acetoxy-3,5-diiodophenoxy)-3,5-diiodophenyl) propanoate #**3** (20 mg, 94%). Nuclear magnetic resonance (NMR) of the product (300 MHz, CDCl_3_) showed the following results: δ 7.61 (s, 2H), 7.18 (s, 2H), 6.02 (d, J = 6.80 Hz, 1H), 4.84 (q, J = 6.69 Hz, 1H), 3.76 (s, 3H), 3.16-2.97 (m, 2H), 2.38 (s, 3H), 2.05 (s, 3H).

**(S)-2-acetamido-3-(4-(4-hydroxy-3,5-diiodophenoxy)-3,5-diiodophenyl)propanoic acid (#4)**

NaOH (5.5 mg, 0.137 mmol) was added to a solution of (S)-2-acetamido-3-(4-(4-acetoxy-3,5-diiodophenoxy)-3,5-diiodophenyl) propanoate #**3** (15 mg, 0.017 mmol) in THF/water (20 mL, 3:1). The reaction mixture was stirred at ambient temperature for 12 h. The THF was removed *in vacuo*, and the resulting solution was acidified with 1N HCl to pH 2–3. More water was added (30 mL), and the aqueous solution was extracted with ethyl acetate (3 × 25 mL). The combined organic layer was washed with brine, dried over sodium sulfate, and concentrated. The residue was purified by silica gel column chromatography to produce (S)-2-acetamido-3-(4-(4-hydroxy-3,5-diiodophenoxy)-3,5-diiodophenyl)propanoic acid #**4** (13 mg, 92%) as a white solid.

**(S)-2-((tert-butoxycarbonyl)amino)-3-(4-(4-hydroxy-3,5-diiodophenoxy)-3,5-diiodophenyl)propanoic acid (#5)**

NaOH (17.96 mg, 0.449 mmol) was added to a solution of methyl (S)-2-((tert-butoxycarbonyl) amino)-3-(4-(4-hydroxy-3,5-diiodophenoxy)-3,5-diiodophenyl)propanoate 2 (100 mg, 0.112 mmol) in THF/water (20 mL, 3:1). The reaction mixture was stirred at ambient temperature for 12 h. The THF was removed *in vacuo*, and the resulting solution was acidified with 1 N HCl to pH 4. More water was added (30 mL), and the aqueous solution was extracted with ethyl acetate (3 × 25 mL). The combined organic layer was washed with brine, dried over sodium sulfate, and concentrated. A crude product was isolated from ethyl acetate/n-hexane to obtain (S)-2-((tert-butoxycarbonyl)amino)-3-(4-(4-hydroxy-3,5-diiodophenoxy)-3,5-diiodophenyl)propanoic acid #**5** (90 mg, 91%) as a white solid.

**Tert-butyl (S)-(1-((3-((tert-butoxycarbonyl)amino)propyl)amino)-3-(4-(4-hydroxy-3,5-diiodophenoxy)-3,5-diiodophenyl)-1-oxopropan-2-yl)carbamate (#6)**

Using the procedure described for #**8** with tert-butyl (3-aminopropyl)carbamate 12 produced compound #**6** with a 75% yield.

**(S)-2-amino-N-(3-aminopropyl)-3-(4-(4-hydroxy-3,5-diiodophenoxy)-3,5-diiodophenyl)propanamide dihydrochloride (#7)**

A 4.0 M hydrogen chloride solution in 1,4 dioxane (1 mL) was added to a mixture of tert-butyl (S)-(1-((3-((tert-butoxycarbonyl)amino)propyl)amino)-3-(4-(4-hydroxy-3,5-diiodophenoxy)-3,5-diiodophenyl)-1-oxopropan-2-yl)carbamate #**6** (25 mg, 0.024 mmol) in ethyl acetate (7 mL), and the mixture was stirred for 12 h. The mixture was concentrated to minimum volume, and the residue was collected by filtration to obtain (S)-2-amino-N-(3-aminopropyl)-3-(4-(4-hydroxy-3,5-diiodophenoxy)-3,5-diiodophenyl)propanamide dihydrochloride #**7** (15 mg, 74%) as an off-white solid.

**Tert-butyl((S)-3-(4-(4-hydroxy-3,5-diiodophenoxy)-3,5-diiodophenyl)-1-oxo-1-(((1R,2S)- 2-phenylcyclopropyl)amino)propan-2-yl)carbamate (#8)**

A mixture of (S)-2-((tert-butoxycarbonyl)amino)-3-(4-(4-hydroxy-3,5-diiodophenoxy)-3,5-diiodophenyl)propanoic acid #**5** (90 mg, 0.103 mmol), EDCI (49.18 mg, 0.257 mmol), trans-2-phenylcyclopropane-1-amine hydrochloride (19.15 mg, 0.113 mmol), hydroxybenzotriazole (20.80 mg, 0.154 mmol), and Hünig's base (46.53 mg, 0.359 mmol) in dichloromethane (10 mL) was stirred for 24 h. The reaction mixture was diluted with aqueous NaHCO_3_ and extracted with dichloromethane. The extracts were washed with brine, dried over anhydrous sodium sulfate, and concentrated *in vacuo*. The residue was purified by silica gel column chromatography to obtain tert-butyl ((S)-3-(4-(4-hydroxy-3,5-diiodophenoxy)-3,5-diiodophenyl)-1-oxo-1-(((trans)-2-phenylcyclopropyl)amino)propan-2-yl)carbamate, compound #**8** (90 mg, 88%). The NMR spectra (400 MHz, CDCl3) results were as follows: δ 7.71 (s, 2H), 7.30 - 7.24 (m, 4H), 7.21 -7.11 (m, 2H), 7.10 (d, J = 2.06 Hz, 2H), 6.20 (d, J = 2.52 Hz, 1H), 5.54 (s, 1H), 5.16 - 5.07 (m, 1H), 3.10 - 2.91 (m, 2H), 2.90 -2.82 (m, 1H), 2.05 - 1.95 (m, 1H), 1.45 (s, 9H), 1.31 - 1.18 (m, 1H), 1.12 -1.02 (m, 1H).

**(S)-2-amino-3-(4-(4-hydroxy-3,5-diiodophenoxy)-3,5-diiodophenyl)-N-((1R,2S)- 2-phenylcyclopropyl)propanamide hydrochloride (#9)**

Hydrogen chloride (4 N) in ethyl acetate (0.5 ml) was added to a solution of tert-butyl ((S)-3-(4-(4-hydroxy-3,5-diiodophenoxy)-3,5-diiodophenyl)-1-oxo-1-(((trans)-2-phenylcyclopropyl)amino)propan-2-yl)carbamate #**8** (90 mg, 0.091 mmol) in ethyl acetate (6 mL) and stirred continuously for 24 h. The reaction mixture was concentrated *in vacuo*, and the residue was collected by filtration to obtain (S)-2-amino-3-(4-(4-hydroxy-3,5-diiodophenoxy)-3,5-diiodophenyl)-N-((trans)-2-phenylcyclopropyl)propanamide hydrochloride #**9** (66 mg, 78%) as a white solid. The 1H NMR (400 MHz, DMSO-d6) spectra results were as follows: δ 9.28 (s, 1H), 8.86 (dd, J = 4.27, 28.69 Hz, 1H), 8.29 (bs, 3H), 7.80 (d, J = 9.16 Hz, 2H), 7.30 - 7.23 (m, 2H), 7.20 - 7.09 (m, 5H), 3.98 - 3.88 (m, 1H), 3.10 - 3.90 (m, 2H), 2.88 - 2.75 (m, 1H), 2.04 - 1.79 (m, 1H), 1.28 - 1.18 (m, 1H), 1.17 - 1.06 (m, 1H).

**SUPPLEMENTARY FIGURES**


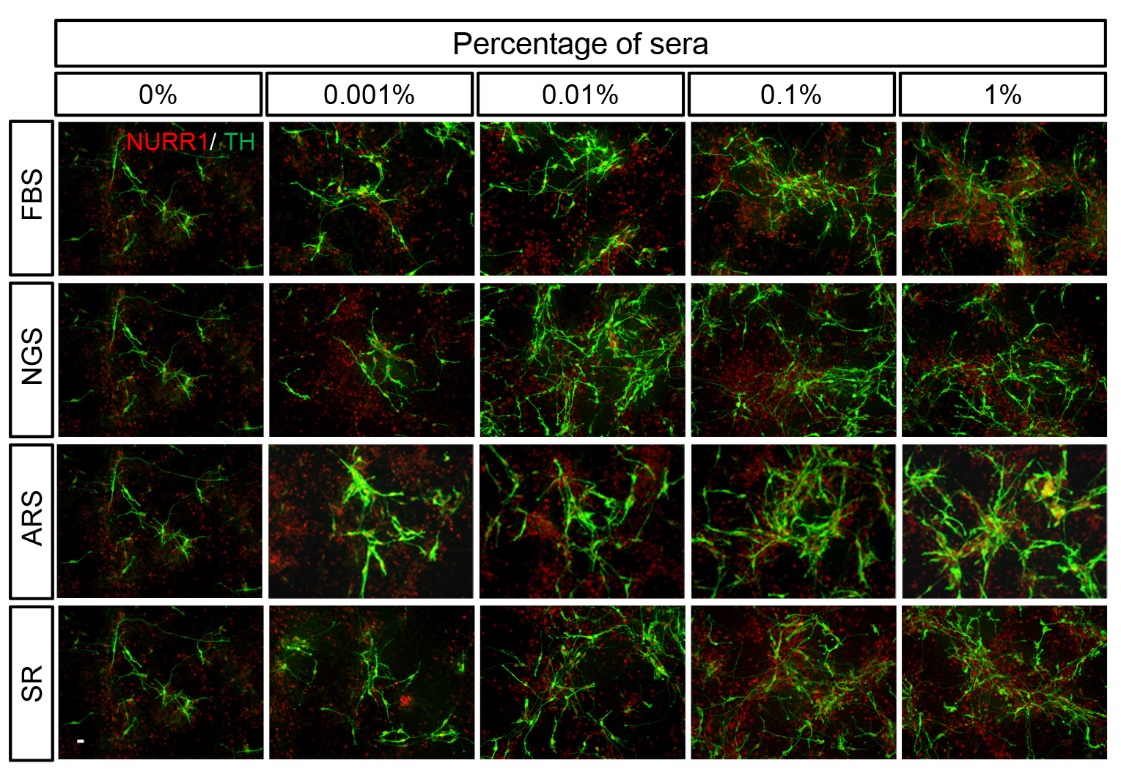


**Supplementary Figure 1. Other sera induce DA neurons similar to FBS**

*Nurr1*-overexpressing rat cortical NPCs were treated with different concentrations of different sera (fetal bovine serum (FBS), normal goat serum (NGS), adult rat serum (ARS) and human serum (HS)) for 7 days. On differentiation day 7, immunostaining was performed to identify NURR1+ and TH+ cells for comparison between the sera. The induction of DA neuron differentiation was concentration-dependent, and the patterns were similar for the different sera. Scale bar, 20 μm.


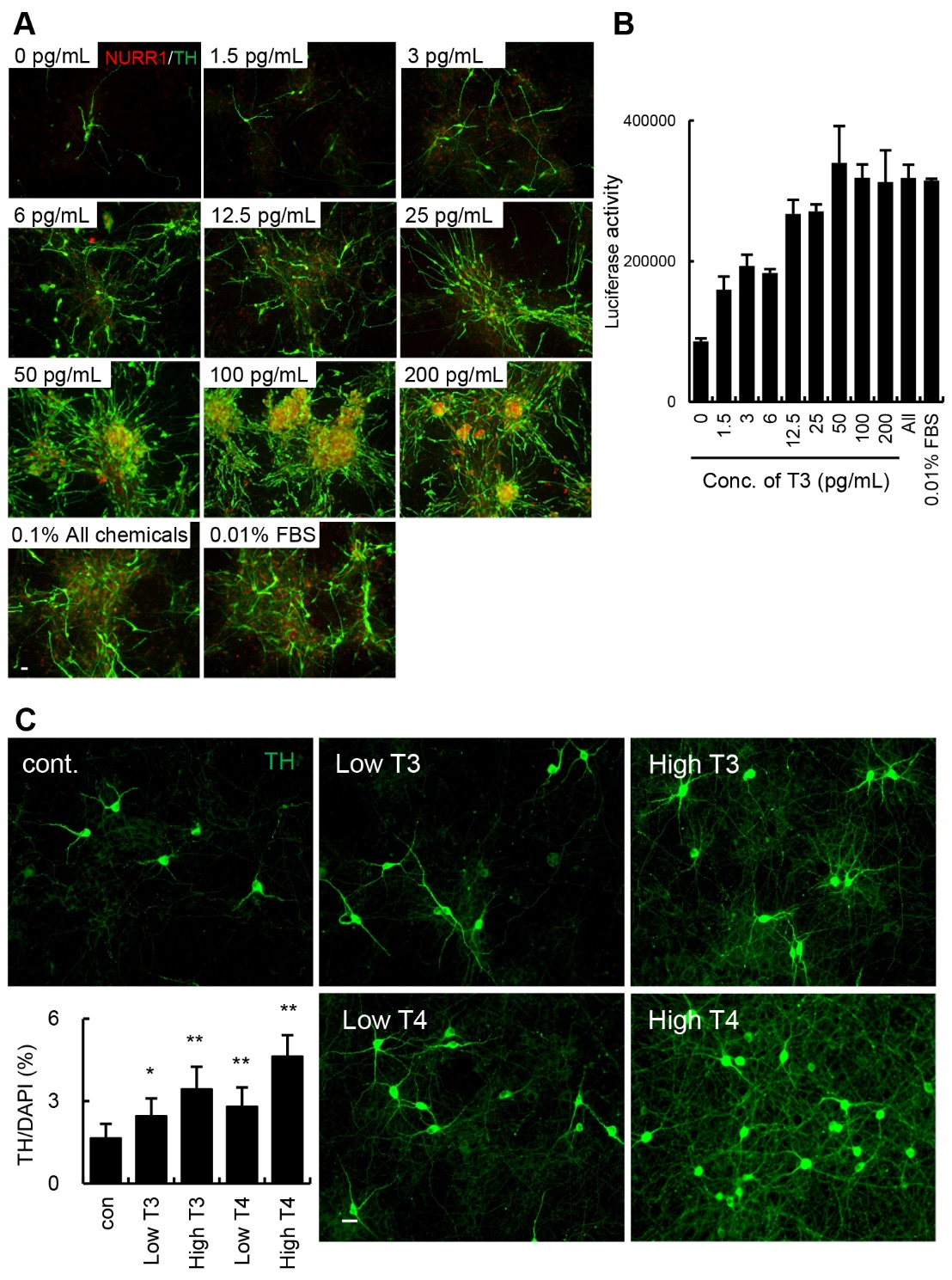


**Supplementary Figure 2. The effects of T3 and T4 are concentration-dependent**

(A) T3 was used to treat *Nurr1-Mash1*-overexpressing rat NPCs at various concentrations. Mixtures of the 17 chemicals found in FBS (0.1% for all chemicals) and 0.01% FBS were administered, and the results were compared with those of the T3 treatment. On differentiation day 7, an immunocytochemical analysis was performed for NURR1+ and TH+ cells. (B) The TH promoter assay showed that TH promoter activity occurred in a concentration-dependent manner. (C) Rat embryonic E14 VM NPCs were treated with T3 and T4 for two weeks. The concentration of "low T3" was 1.5 pg/mL; the concentration of "high T3" was 50 pg/mL; the concentration of "low T4" was 0.15 pg/mL; and the concentration of "high T4" was 1.5 pg/mL. The number of TH+ cells increased following treatment with the thyroid hormones, and the effect occurred in a concentration-dependent manner. The error bars are the S.E. *p < 0.05, **p < 0.01. Scale bar, 20 μm.

**Supplementary Figure 3. Synthesis of thyroid hormone derivatives**

The chemical structures of the nine thyroid hormone derivatives produced by the Korea Research Institute of Chemical TechnologyKorea Research Institute of Chemical Technology (KRICT)

.


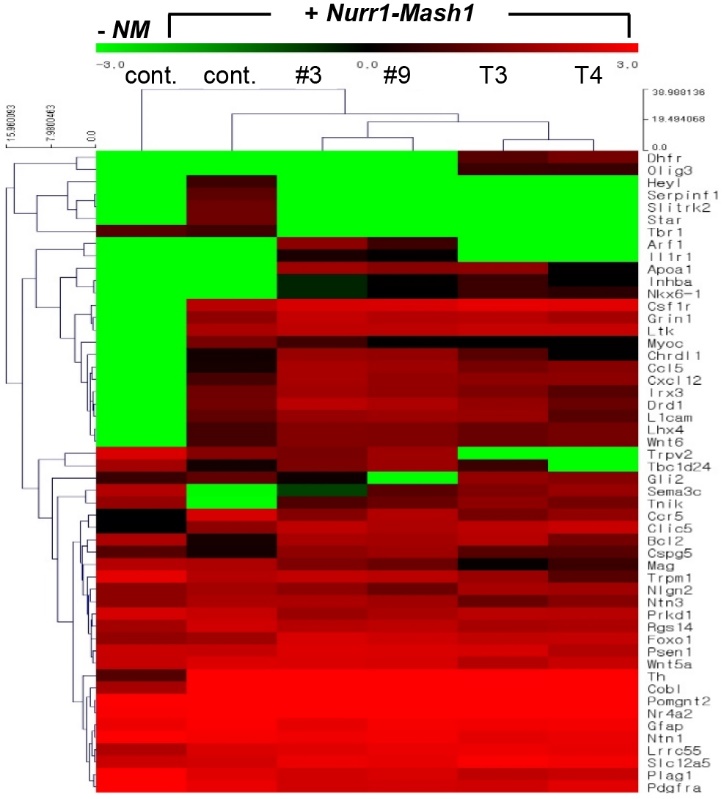


**Supplementary Figure 4. Heat map of rat *Nurr1-Mash1*-overexpressing NPCs treated with each chemical on differentiation day 2**

The data were normalized to gene expression in nonviral-infected cells. Red, upregulated; green, downregulated. The bar at the top represents the log2 transformed values.


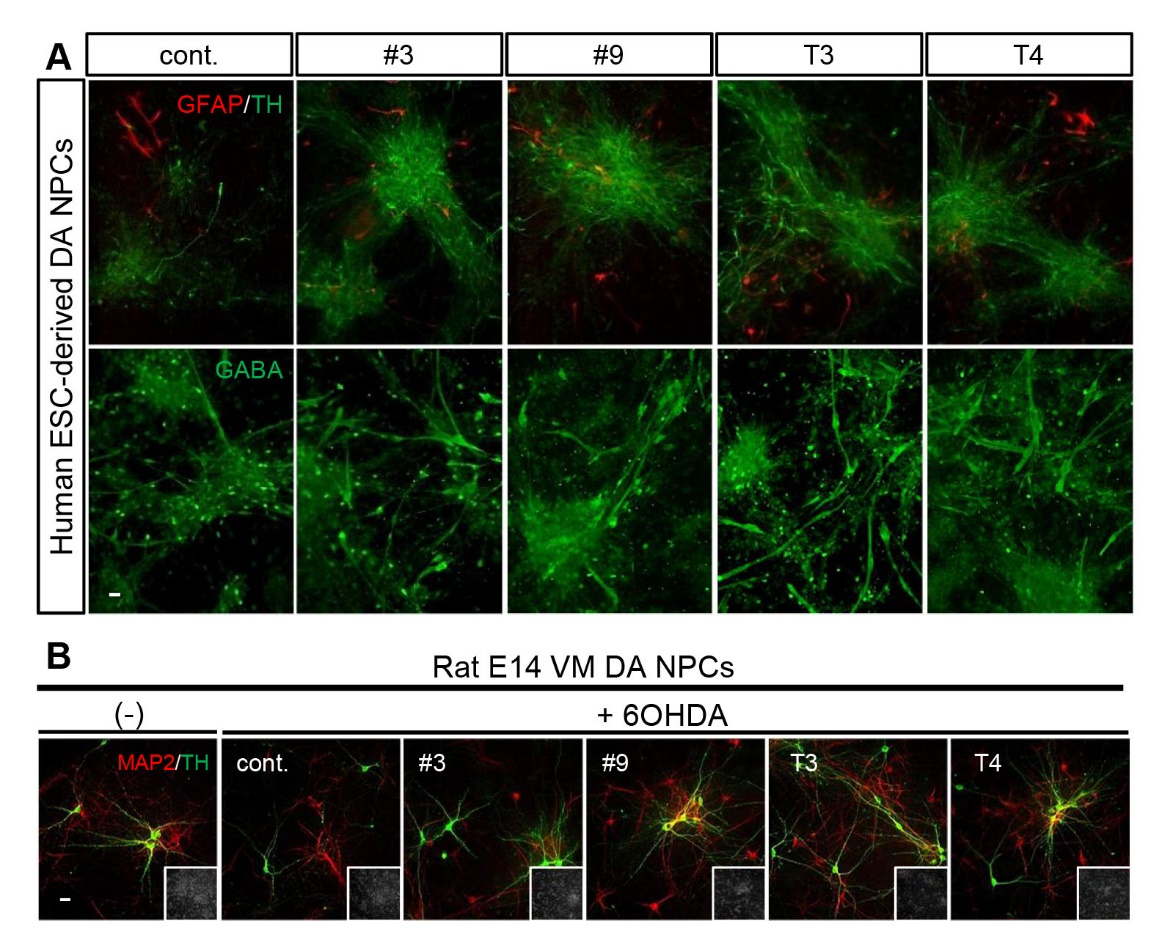


**Supplementary Figure 5. The effect of derivatives is specific to DA neurons but not other neural lineage cells**

(A) Human ESC-derived DA NPCs treated with thyroid hormones and their derivatives were differentiated into DA neurons. Anti-GFAP and anti-GABA antibodies were used as other neural lineage cell markers. (B) Rat VM NPCs were damaged by 6-OHDA (18 h) and differentiated with thyroid hormones and derivatives for 14 days. Anti-MAP2 antibody was used as a mature neuronal marker, including non-DA neurons. Scale bar, 20 μm.

**SUPPLEMENTARY TABLES**

**Supplementary Table 1. The composition of purchased FBS^12^**

| **Component** | **Concentration** |
| --- | --- |
| 1. Cortisol (Sigma-Aldrich #C-106) | 0.5 ng/mL |
| 2. Growth hormone (Sigma-Aldrich #H5916) | 39 ng/mL |
| 3. Parathyroid hormone (PTH) (Sigma-Aldrich #P7036) | 1.72 ng/mL |
| 4. Triiodothyronine (T3) (Sigma-Aldrich #T6397) | 1.2 ng/mL |
| 5. Thyroxine (T4) (Sigma-Aldrich #T1775) | 0.12 ng/mL |
| 6. Follicle-stimulating hormone (FSH) (Sigma-Aldrich #F4021) | 0.095 ng/mL |
| 7. Luteinizing hormone (LH) (Sigma-Aldrich #L6420) | 0.008 ng/mL |
| 8. Prostaglandin E1 (Sigma-Aldrich #P7527) | 6 ng/mL |
| 9. Prostaglandin E2 (Sigma-Aldrich # P6532) | 6 ng/mL |
| 10. Prostaglandin F1a (Sigma-Aldrich #P5765) | 12.3 ng/mL |
| 11. Retinoic acid 9-cis (Sigma-Aldrich #R4643) | 90 ng/mL |
| 12. Retinoic acid all-trans (Sigma-Aldrich #R2625) | 90 ng/mL |
| 13. Cholesterol (Sigma-Aldrich #C3045) | 310000 ng/mL |
| 14. Lactate dehydrogenase (LDH) (Sigma-Aldrich # LLDH-RO) | 864 mU/mL |
| 15. Aspartate aminotransferase (ASAT) (Sigma-Aldrich # G2751) | 130 mU/mL |
| 16. Alkaline phosphatase (Sigma-Aldrich #APMB-RO) | 255 mU/mL |
| 17. Thyroid-stimulating hormone (TSH) (Sigma-Aldrich #T9265) | 1.22 ng/mL |

**Supplementary Table 2. Primers for RT-PCR**

**Rat**

| **Gene**  **Name** | **Primer Sequences** | **Annealing**  **Temperature** | **Cycle Number** | **PCR Product** |
| --- | --- | --- | --- | --- |
| *TH* | TCAAGACTGACTCACAGCAACCCC | 60℃ | 25 cycles | 412 bp |
|  | CTTTGTCCTGAACCGTGGTGGTAG |  |  |  |
| *Nurr1* | CGGTTTCAGAAGTGCCTAGC | 58℃ | 23 cycles | 214 bp |
|  | TTGCCTGGAACCTGGAATAG |  |  |  |
| *DAT* | GCTGGCACATCTATCCTCTTTGG | 60℃ | 25 cycles | 182 bp |
|  | CAATGCTGACCACGACCACATAC |  |  |  |
| *AADC* | GCCTTTATCTGTCCTGAGTTCCG | 60℃ | 25 cycles | 198 bp |
|  | TGATGAGTCCTGAGTCCTGGTGAC |  |  |  |
| *GAPDH* | GGCATTGCTCTCATTGACAA | 60℃ | 23 cycles | 165 bp |
|  | AGGGCCTCTCTCTTGCTCTC |  |  |  |

**Human**

| **Gene**  **Name** | **Primer Sequences** | **Annealing**  **Temperature** | **Cycle Number** | **PCR Product** |
| --- | --- | --- | --- | --- |
| *TH* | GAGTACACCGCCGAGGAGATTG | 58℃ | 27 cycles | 279 bp |
|  | GCGGATATACTGGGTGCACTGG |  |  |  |
| *Nurr1* | TTCTCCTTTAAGCAATCGCCC | 60℃ | 30 cycles | 332 bp |
|  | AAGCCTTTGCAGCCCTCACAG |  |  |  |
| *En1* | GCAACCCGGCTATCCTACTTATG | 60℃ | 30 cycles | 247 bp |
|  | ATGTAGCGGTTTGCCTGGAAC |  |  |  |
| *Lmx1b* | ACGAGGAGTGTTTGCAGTGCG | 60℃ | 27 cycles | 253 bp |
|  | CCCTCCTTGAGCACGAATTCG |  |  |  |
| *GAPDH* | GCTCAGACACCATGGGGAAGGT | 58℃ | 27 cycles | 474 bp |
|  | GTGGTGCAGGAGGCATTGCTGA |  |  |  |
